# Supplementary material for: Assessing Face Validity of the HexCom Model for Capturing Complexity in Clinical Practice: A Delphi Study
Source: Healthcare (Basel). 2021 Feb 4;9(2):165. doi: 10.3390/healthcare9020165 (PMC7913893; doi:10.3390/healthcare9020165)
Supplement: Supplementary file 1 [file healthcare-09-00165-s001.zip › Supplementary materials HexCOM1.pdf]

*HexCom®-Red*2019

Model for the care of people with advanced disease and/or end of life situation

Reduced version for the management and referral of complex cases.
OBSERVED CARE COMPLEXITY.

NEEDS:
Identify the patient's areas of **discomfort** and relate it to the possibility of **response** from the service.

Mark the level of complexity of the affected areas: ***L Low, M Medium, H High.*** *Mark* ***N*** *for areas Not evaluated / Not applicable.*

COMPLEXITY AND INTERPRETATION LEVELS:
L - Low (little difficulty). Guarantees of being able to attend to the situation with the resources of the service.
M - Medium (moderate difficulty). Guarantees of taking on the situation with the support of other professionals and/or specialized teams.
H - High (refractory difficulty). Little chance of change. It is necessary to escort and/or probably refer to another resource/level of care.

**CLINIC**	Physical discomfort due to symptoms (pain, dyspnea), refractory lesions (malignant ulcer), or therapeutic difficulty (adherence or access to drugs/techniques).	
**PSYCHO-EMOTIONAL**	Emotional distress of maladaptive type (intense, persistent emotions that interfere with relationships and functionality), rigid personality traits (unable to adapt), psycho-pathological profile.	
**SPIRITUAL**	Deep distress with a feeling of biographical rupture, lack of meaning (personal, vital, suffering), loneliness (unwanted, isolation from relational rupture), feelings of guilt, inability to forgive (oneself, others), panic over what is to come (death itself or the separation of loved ones), feeling of injustice.	
**SOCIAL AND FAMILY**	Relational distress caregiver-patient, insufficient care, no resources, no caregiver.	
**ETHICS**	Upset about information management, clinical decisions / therapeutic effort adaptation (TEA), desire to advance death (DAD).	
**DIRECT RELATIONSHIP WITH DEATH / DYING PROCESS**	Distress due to problematic location of death. Maladaptive denial of the situation in the last days situation (LDS), traumatic LDS, difficult sedation (by practice, indication, understanding). Risk factors for complicated bereavement.	
	**COMPLEXITY LEVEL:** The highest observed in any of the affected areas:	

INTERVENTION CRITERIA FOR REFERENCE TEAM - SPECIALIZED TEAM ACCORDING TO THE COMPLEXITY LEVEL

L - Low: Intervention of the reference team and on occasion the specialized team.
M - Medium: Intensity shared care agreed by both teams.
H - High: Intense intervention by the specialized team. Probable referral to another resource or level of care.

*HexCom®-Basic*2019

Model for the care of people with advanced disease and/or end of life situation.

Basic version for the assessment of needs and strengths.
OBSERVED CARE COMPLEXITY.

NEEDS:
Identify the patient's areas of discomfort and relate it to the possibility of response from the service.

Mark the level of complexity of the affected areas: ***L Low, M Medium, H High.*** *Mark* ***N*** *for areas Not evaluated / Not applicable.*

COMPLEXITY AND INTERPRETATION LEVELS:
L - Low (little difficulty). Guarantees of being able to attend to the situation with the resources of the service.
M - Medium (moderate difficulty). Guarantees of taking on the situation with the support of other professionals and/or specialized teams.
H - High (refractory difficulty). Little chance of change. It is necessary to escort and/or probably refer to another resource/level of care.

**CLINIC**	Physical discomfort due to symptoms (pain, dyspnea), refractory lesions (malignant ulcer), or therapeutic difficulty (adherence or access to drugs/techniques).	
**PSYCHO-EMOTIONAL**	Emotional distress of maladaptive type (intense, persistent emotions that interfere with relationships and functionality), rigid personality traits (unable to adapt to changes), psycho-pathological profile.	
**SPIRITUAL**	Deep distress with a feeling of biographical rupture, lack of meaning (personal, vital, suffering), loneliness (unwanted, isolation from relational rupture), feelings of guilt, inability to forgive (oneself, others), panic over what is to come (death itself or the separation of loved ones), feeling of injustice.	
**SOCIAL AND FAMILY**	Relational distress caregiver-patient, insufficient care, no resources, no caregiver.	
**ETHICS**	Upset about information management, clinical decisions / therapeutic effort adaptation (TEA), desire to advance death (DAD).	
**DIRECT RELATIONSHIP WITH DEATH / DYING PROCESS**	Distress due to problematic location of death. Maladaptive denial of the situation in the last days situation (LDS), traumatic LDS, difficult sedation (by practice, indication, understanding). Risk factors for complicated bereavement.	
	**COMPLEXITY LEVEL:** The highest observed in any of the affected areas:	

STRENGTHS:

Identifies the systemic source of **strength** and relates it to the possibility of **empowerment** by the service.

System: The person understood as an integral and interacting element of five systems, which interact with each other and with the blurred boundaries. Metaphorical example: the flight of starlings.

*Mark the level of strength of the systems: **L Low, M Medium, H High.** Mark **N** for areas Not evaluated / Not applicable.*

STRENGTH AND INTERPRETATION LEVELS:

L - Low (absent): Lacking strength or it is not used. High difficulty to be able to empower.
M - Medium (precarious): Fragile strain that must be validated, preserved and enhanced in a preferential way.
H - High (consistent): Firm support that probably will not change in the course of the disease.

**MICRO-SYSTEM** (individual)	The strength of the patient and/or family caregivers (values, mental and physical capacity, resilience, adaptability, preferences, decision making, extensive network...).	
**MESO-SYSTEM** (interactions)	The strength of the bond with family and/or community members (tone of care, organizational capacity...); and/or with the referring professionals; and/or economic strength.	
**CRONO-SYSTEM** (time)	Having pending issues ordered (legacy, economy, inheritance, links...); and/or to anticipate a progressive course of the disease, without sudden changes, and without changes in the care environment.	
**EXO-SYSTEM** (team)	The strength and competence of the team to meet the needs of patients/families (interdisciplinarity, training, communication, community integration, continuity, and consensus on the therapeutic objective).	
**MACRO-SYSTEM** (resources)	The sufficient provision and accessibility to services and/or resources to meet the needs of the patient (care continuity, home help service, dependency law, residential, hospital or social and healthcare admission, radiotherapy units, pain clinic...).	
	**STRENGTH LEVEL:** Number of systems with high strength (H):	
	**BALANCE OF COMPLEXITY:** complexity level +/- strength level:<br><br>*Intuitive balance between needs and strengths: In principle, consider whether more than half of the systems with high strength (H) could reduce the level of final complexity by one; or conversely, augment it if there is no strength in more than half of the systems.*	

INTERVENTION CRITERIA FOR REFERENCE TEAM - SPECIALIZED TEAM ACCORDING TO THE COMPLEXITY BALANCE

L - Low: Intervention of the reference team and on occasion the specialized team.
M - Medium: Intensity shared care agreed by both teams.
H - High: Intense intervention by the specialized team. Probable referral to another resource or level of care.

HexCom®-Clin 2019

Model for the care of people with advanced disease and/or end of life situation.

Extended version for the assessment of needs and resources.

OBSERVED CARE COMPLEXITY.

NEEDS:

Identify the patient's areas of discomfort and relate it to the possibility of response from the service.

Mark the level of complexity of the affected areas: ***L Low, M Medium, H High.*** *Mark* ***N*** *for areas Not evaluated / Not applicable.*

COMPLEXITY AND INTERPRETATION LEVELS:

L - Low (little difficulty). Guarantees of being able to attend to the situation with the resources of the service.

M - Medium (moderate difficulty). Guarantees of taking on the situation with the support of other professionals and/or specialized teams.

H - High (refractory difficulty). Little chance of change. It is necessary to escort and/or probably refer to another resource/level of care.

**Area**	**Sub-area**		N	L	M	H
**CLINIC**	PHYSICAL	Physical discomfort due to symptoms (pain, dyspnea...) and/or injuries (tumorous ulcer...).				
	THERAPEUTICS	Difficulty in adherence to prescriptions and/or access to drugs/techniques.				
**PSYCHO-EMOTIONAL**	PERSONALITY	Psychological vulnerability: rigid personality traits with difficulty adapting to changes (perfectionism, thoroughness, control...), or psychopathology (enolism, drug addiction, psychiatric disease, dementia with behavioural disturbance, delirium...).				
	EMOTIONAL	Maladaptive emotional distress (intense, persistent, interfering with relationships and functionality).				
**SPIRITUAL**	SENSE	Deep distress with feelings of rupture due to illness, with difficulty finding meaning in the situation, feelings of incoherence with the actions and decisions taken throughout life.				
	CONNECTION	Deep distress with isolation and rupture of relationships, feelings of guilt, does not feel at peace with others or that they are one of them, difficulty forgiving, inability to use insight.				
	TRANSCENDENCE	Deep distress with difficulty facing everything that will come, in the face of the unknown: panic at dying, at disappearing, the future of those left, difficulty seeing what they will leave, feelings of injustice.				
**SOCIAL AND FAMILY**	RELATIONAL	Relational distress in the family environment that makes patient care difficult.				
	EMOTIONAL	Emotional maladjustment of the caregiver/s (intense, persistent, hindering relationships and functionality) and which makes patient care difficult.				
	PRACTICE	Distress due to difficulty in managing the basic needs of the patient (hygiene, food, safety...).				
	EXTERNAL	Distress due to the lack of effective external support for the cohabiting nucleus.				
	MONEY	Financial distress and/or difficulties in hiring external help and/or accessing resources.				
**ETHICS**	INFORMATION	Distress due to difficulties in the management of information concerning diagnosis and/or prognosis.				
	CLINICAL DECISIONS	Difficulties in clinical decision making (adequacy of diagnostic and/or therapeutic effort).				
	DESIRE TO ADVANCE DEATH	Desire to advance death (DAD) in any degree: thought, intention, decision, plan and/or explicit request.				
**DIRECT RELATIONSHIP WITH DEATH / DYING PROCESS**	LOCATION	Difficulties in planning the place to die (no agreement between patient-caregiver) or request to change location.				
	SITUATION IN THE LAST DAYS (LDS)	Difficulties in managing the dying process (maladaptive denial of the situation, refractory symptoms, difficult sedation).				
	MOURNING	Risk factors during mourning.				

**COMPLEXITY LEVEL:** The highest observed in any of the affected sub-areas:	

STRENGTHS:

Identifies the systemic source of **strength** and relates it to the possibility of **empowerment** by the service.

System: The person understood as an integral and interacting element of five systems, which interact with each other and with the blurred boundaries. Metaphorical example: the flight of starlings.

*Mark the level of strength of the systems: **L Low, M Medium, H High.** Mark **N** for areas Not evaluated / Not applicable.*

STRENGTH AND INTERPRETATION LEVELS:

L - Low (absent): Lacking strength or it is not used. High difficulty to be able to empower.
M - Medium (precarious): Fragile strain that must be validated, preserved and enhanced in a preferential way.
H - High (consistent): Firm support that probably will not change in the course of the disease.

**System:**		**Sub-system:**	N	L	M	H
**MICRO-SYSTEM**	Individuals: The patient and their caregivers	The patient's strength (values, mental capacity, resilience/adaptability, preferences/priorities).				
		The strength of family caregivers (values, mental/physical capacity, resilience/adaptability, extended network).				
**MESO-SYSTEM**	Interactions: Interaction of the patient with the relational network and the professional network	The strength of family bonds and/or community bonds (tone of care, organizational capacity...).				
		The strength of the bonds with the professionals concerned (doctor/nurse, specialist).				
		Economic strength.				
**CHRONO-SYSTEM**	Time: Changes in time	Having ordered pending issues that generate concern (legacy, economy, inheritance, bonds...).				
		Anticipating a progressive course of the disease, without sudden changes, and without changes in the care environment.				
**EXO-SYSTEM**	Team: Competence of the team	The strength and competence of the team to meet the needs of the patients/family (interdisciplinarity, training, communication, community integration, continuity, and consensus on the therapeutic objective).				
**MACRO-SYSTEM**	Resources: Availability of health, and social and healthcare services/resources	The sufficient provision and accessibility to social and healthcare services and/or resources to meet the needs of the patient (care continuity, home help service, dependency law, residential places, hospital or social and healthcare admission, radiotherapy units, pain clinic, day hospital...).				
		**STRENGTH LEVEL:** Number of sub-systems with high strength (H):				

**BALANCE OF COMPLEXITY:** complexity level +/- strength level:	

Intuitive balance between needs and strengths: In principle, consider whether more than half of the sub-systems with high strength (H) could reduce the level of final complexity by one; or conversely, augment it if there is no strength in more than half of the systems.	

INTERVENTION CRITERIA FOR REFERENCE TEAM - SPECIALIZED TEAM ACCORDING TO THE COMPLEXITY BALANCE

L - Low: Intervention of the reference team and on occasion the specialized team.
M - Medium: Intensity shared care agreed by both teams.
H - High: Intense intervention by the specialized team. Probable referral to another resource or level of care.

HexCom®-Patient2019

Model for the care of people with advanced disease.

Guided interview version.

SELF-REFERRED CARE COMPLEXITY.

"Given the situation you are going through, it would help us to know how you are experiencing it. That's why we would like to ask you some questions, if that's okay for you."

NOTE 1: Consider first the need to create a good bond and respond to emerging needs.
NOTE 2: Ask the questions sitting, in a protected place, in an empathic context, with eye contact, and giving time for active listening as long as is necessary.
NOTE 3: Seek to guarantee the therapeutic effect of the interview itself. Assess whether to interview patients completely or only in part in patients presenting psycho-emotional complexity (e.g. maladaptive denial or refractory suffering, confused state or cognitive impairment). In these cases, assess whether the main caregiver can answer for the patient.

**NEEDS:**		Level of distress		
		**L** Low	**M** Medium	**H** High
**CLINICAL**	Do you currently have pain, shortness of breath, or other symptoms...? If so, to what degree do you feel unwell?			
**PSYCHO-EMOTIONAL**	Currently, do you have emotional distress (sadness, anxiety or nervousness, anger, insomnia...)? If so, to what degree?			
**SPIRITUAL**	Do you currently feel broken down by the disease, without a meaning in life, and/or do you feel relationships have broken with those you love, and/or have feelings of injustice or fear for what may happen? If so, to what extent do these feelings cause you distress?			
**SOCIAL AND FAMILY**	Do you currently have difficulties in the relationship with your family caregivers, and/or do you feel unattended, or do you see that "there are not enough hands" or resources...? If so, to what degree does it bother you?			
**ETHICS**	Are you currently worried about not having enough information to participate in decision making? If so, to what degree does it bother you?			
**DIRECT RELATIONSHIP WITH DEATH OR WITH THE DYING PROCESS**	Given the possibility that things are not going well, are you worried about the way you may be taken care of, the place where you want to be, and/or the grief of your family? If so, to what degree does it bother you?			

PASSING OF TIME

"In general, how would you say time is passing, does it appear fast or slow?"	Normal/Fast	Slow	Very slow

"What would you relate it to?"

(Take into consideration that sometimes the question about the passage of time discriminates the capacity for diversification of attention and sources of gratification more than the suffering of the patient)

**RESOURCES**		Strength level		
		**L**<br>Low	**M**<br>Plenty	**H**<br>High
**MICRO-SYSTEM**<br>(individuals)	To what degree does your internal strength help you sustain the current situation (your values, adaptability/resilience, firmness in decision making or priorities...) and/or the strength of your family caregivers?			
**MESO-SYSTEM**<br>(interactions)	To what degree does the affection and the relationship with your relatives, caregivers, and/or with the professionals concerned, and/or economic strength help you to sustain the current situation?			
**CHRONO-SYSTEM**<br>(time)	To what degree are you satisfied with what you have contributed to your loved ones, to life or to the world and/or to what extent do you think any pending issues could be resolved?			
**EXO-SYSTEM**<br>(team)	To what degree do you feel well attended and safe with the care you receive from the team?			
**MACRO-SYSTEM**<br>(resources)	To what degree do you consider that you have the appropriate social and healthcare resources available to deal with the situation?			
"What helps you most in the current situation?"				
**BALANCE**: "So, now, taking stock of the concerns and strengths you show us, the priority for you would be...":				
