# Supplementary figures and images for: Assessing Face Validity of the HexCom Model for Capturing Complexity in Clinical Practice: A Delphi Study
Source: Healthcare (Basel). 2021 Feb 4;9(2):165. doi: 10.3390/healthcare9020165 (PMC7913893; doi:10.3390/healthcare9020165)

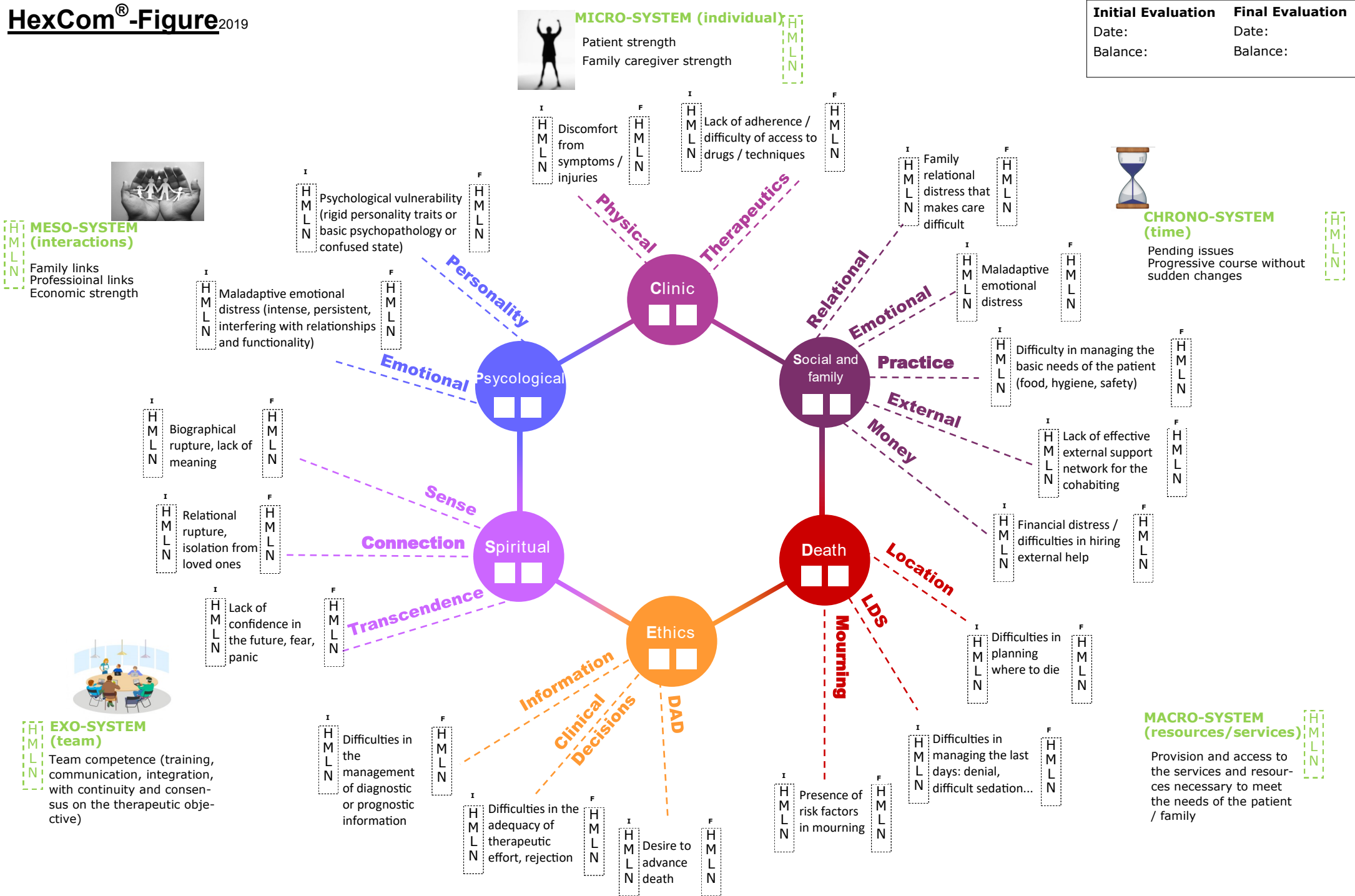

Supplement: Supplementary file 1 [file healthcare-09-00165-s001.zip › Supplementary materials HexCOM2.pdf]
